# Evaluation of Ovarian Reserve with Anti-Müllerian Hormone in Familial Mediterranean Fever

**DOI:** 10.1155/2015/380354

**Published:** 2015-05-12

**Authors:** Ali Şahin, Savaş Karakuş, Yunus Durmaz, Çağlar Yıldız, Hüseyin Aydın, Ahmet Kıvanç Cengiz, Duygu Güler

**Affiliations:** ^1^Department of Internal Medicine, Rheumatology, Cumhuriyet University Faculty of Medicine, 58140 Sivas, Turkey; ^2^Department of Obstetrics and Gynecology, Cumhuriyet University Faculty of Medicine, Sivas, Turkey; ^3^Department of Physical Medicine and Rehabilitation, Rheumatology, Cumhuriyet University Faculty of Medicine, Sivas, Turkey; ^4^Department of Biochemistry, Cumhuriyet University Faculty of Medicine, Sivas, Turkey

## Abstract

*Objective*. To investigate ovarian reserves in attack-free familial Mediterranean fever (AF-FMF) patients at the reproductive age by anti-Müllerian hormone (AMH), antral follicle count (AFC), ovarian volume, and hormonal parameters. *Methods*. Thirty-three AF-FMF patients aging 18–45 years and 34 healthy women were enrolled and FSH, LH, E2, PRL, and AMH levels were measured in the morning blood samples at 2nd–4th days of menstruation by ELISA. Concomitant pelvic ultrasonography was performed to calculate AFC and ovarian volumes. *Results*. In FMF patient group, median AMH levels were statistically significantly lower in the M69V mutation positive group than in the negative ones (*P* = 0.018). There was no statistically significant difference in median AMH levels between E148Q mutation positive patients and the negative ones (*P* = 0.920). There was also no statistically significant difference in median AMH levels between M680I mutation positive patients and the negative ones (*P* = 0.868). No statistically significant difference was observed in median AMH levels between patients who had at least one mutation and those with no mutations (*P* = 0.868). We realized that there was no difference in comparisons between ovarian volumes, number of follicles, and AMH levels ovarian reserves when compared with FMF patients and healthy individuals. *Conclusions*. Ovarian reserves of FMF pateints were similar to those of healthy subjects according to AMH. However, AMH levels were lower in FMF patients with M694V mutation.

## 1. Introduction

Familial Mediterranean fever (FMF) is an autosomal recessively inherited autoinflammatory disease, which is characterized by attacks of recurrent fever and inflammation of serosal membranes such as peritonitis, pleuritis, pericarditis, and synovitis [1]. In FMF, abdominal pain due to serosal inflammation is the most commonly encountered clinical sign after arthritis-arthralgia and fever [1, 2]. Additionally, erysipelas like erythema is another sign, which may be defined as a specific sign for this disease. It is known that subclinical inflammation continues between attacks in FMF patients [2].

Mutations of the Mediterranean fever (MEFV) gene coding pyrin (marenostrin) protein, which is located in the short arm of the 16th chromosome (16p13.3), are responsible for the disease [2]. More than 150 mutations have been reported in the MEFV gene [2]. The MEFV gene is composed of 10 exons. Most mutations shown to cause the disease are located on the second and 10th exons. The most important mutations located on the 10th exon are M694V, M694I, M680I, and V726A. It has been shown in populations with high disease incidences that the most common mutation is E148Q mutation located in the second exon. It has also been reported that mutations such as A744S and R761H are located on the 10th exon; F479L is located on the 5th exon; P369S is located on the 3rd exon, and some clinical signs may be associated with the disease [2].

Pyrin is a protein made up of 781 amino acids, and it is expressed by neutrophils, eosinophils, monocytes, dendritic cells, and fibroblasts. It has been shown that pyrin regulates nuclear factor kappa-beta (NF-*κ*B) activation, production of potent pyrogenic cytokine, IL-1*β*, and apoptosis [2]. Due to uncontrolled IL-1*β* production, equilibrium and regulation of neutrophil activation are damaged; neutrophil activation is facilitated; and, as a result, a classical FMF attack may develop due to mutation in the pyrin gene.

The only effective current treatment for FMF is colchicine. The most important cause of morbidity and mortality in untreated or inadequately treated FMF patients is amyloidosis [2]. Additionally, considering the age group during which FMF is encountered, the most important concern is the effect of disease on fertility [2].

The correlation between FMF and fertility/infertility is not yet clear [2]. The most important problems reported in female FMF patients have been abortions or early delivery due to synechia caused by peritonitis attacks and uterus contractions caused by peritonitis and fever in pregnant FMF patients [2]. Additionally, possible teratogenic effects of colchicine, which cannot be stopped and should be used for life in the majority of the patients, are still debatable issues [2].

Anti-Müllerian hormone (AMH) is a glycoprotein, which is a member of transforming growth factor-beta family, and it shows its biologic effects which are produced by granulosa cells of follicles at the early developmental stage in ovaries, on transmembrane serine/threonine kinase II receptor (AMHR II) [3, 4]. In previous studies, it was reported that AMH levels were more sensitive in indicating ovarian reserves, and, as the number of follicles decreased with increasing age, AMH levels also decreased [4]. In recent years, AMH has again been identified as a helper marker in the follow-up of ovarian reserves in women with rheumatic diseases [5–7]. However, we did not come across any study in the literature evaluating ovarian reserves in female FMF patients with AMH levels.

The aim of the present study was to investigate ovarian reserves of female FMF patients, who were in the attack-free periods (AFP) and at the reproductive (fertile) age, by AMH, number of antral follicles, ovarian volume, and hormonal parameters. Additionally, it was aimed at comparing ovarian reserves and AMH levels with parameters such as height, weight, body mass index (BMI), history of drug use, amyloidosis, disease severity, and MEFV gene mutations in patients with FMF.

## 2. Materials and Methods

A total of 33 FMF patients fulfilling the Tel Hashomer criteria [8] with AFP and 34 healthy subjects were included in this study. The study protocol was approved by the local ethics committee (dated 13.05.2014 and with decision issue number 2014-05/02) and was in accordance with the Declaration of Helsinki. Informed consent was taken from all participants.

FMF patients who were between 18 and 45 years old and were in the attack-free period (AFP) and healthy female individuals (a lot of them from hospital staff) without any underlying diseases were included in the study. Inclusion criteria included regular menstruation (cycles of 21–35 days), presence of both ovaries, and not receiving treatments other than colchicine (such as biological agents). Exclusion criteria for both groups were premature menopause, chronic liver and kidney diseases, malignancy, history of infertility, smoking, presence of other rheumatic/autoimmune diseases, other gynecological abnormalities (such as polycystic ovarian syndrome, surgical history, history of uterine bleeding, and receiving drug/hormone or similar treatments in the last 3 months), and history of chemotherapy and/or radiotherapy.

While follicle-stimulating hormone (FSH), luteinizing hormone (LH), and estradiol (E2) and prolactin (PRL) levels were measured routinely in venous blood samples obtained in the morning between cycle days 2 and 4 (early follicular phase of the menstrual cycle) using the ELISA method, AMH levels were measured in 5 mL venous blood drawn specifically for the present study by ELISA method.

AMH levels were determined using the AMH/MIS enzyme-linked immunosorbent assay kit (Diagnostic Systems Lab, Webster, Texas, USA). FSH, LH, E2, and PRL were assayed by Chemiluminescent Microparticle Immunoassay (Architect Abbott Lab, IL, USA). Erythrocyte sedimentation rate (ESR) was evaluated by Kimased Auto 60-Vacutest Kima system. C-reactive protein (CRP) was determined by the BN II Nephelometer, Dade Behring (Siemens Healthcare Diagnostics Inc., Deerfield, IL, USA).

The number of antral follicles and ovarian volume were calculated by pelvic ultrasonography performed on the same day. Ovarian volume was measured using the equation of an ellipsoid (length × width × height × 0.526).

Other data such as height, weight, body mass index, history of drug use, drug compliance, presence of amyloidosis, disease severity, monthly attack frequency, family history, number of children (if married), other laboratory parameters, and MEFV mutations were obtained from patient medical records. All FMF patients were under treatment with colchicine (1–1.5 gr daily).

### 2.1. Statistical Analysis

Data analysis was performed by using the SPSS for Windows, version 14.0 (SPSS Inc., Chicago, IL, United States). Whether the distributions of continuous and metric discrete variables were normal or were not determined by Kolmogorov Smirnov test, continuous and metric discrete variables were shown as mean ± standard deviation (SD) or median (min-max), where applicable. Categorical data were expressed as the number of cases and as percentage. While the mean differences between the groups were compared by Student's *t*-test, the Mann Whitney *U* test was applied for the comparisons of median values. Degrees of association between continuous and metric discrete variables were calculated by Spearman rank correlation analyses. A *P* value less than 0.05 was considered statistically significant.

## 3. Results

There was no statistically significant difference in mean age; median number of gravidity, parity, abortions, and children alive; and the mean BMI levels and median number of siblings between the control and FMF groups (*P* > 0.05) (Table 1).

**Figure 1:**
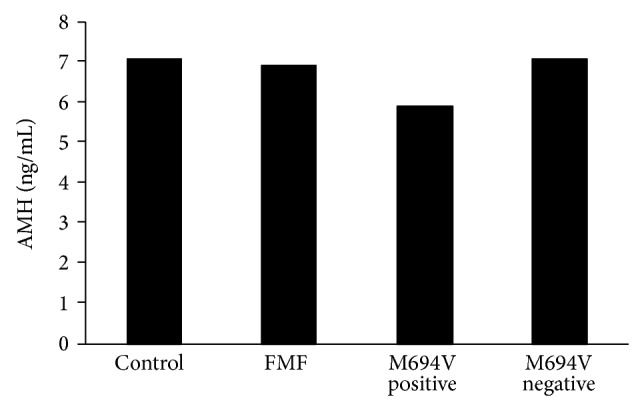
Anti-Müllerian hormone levels according to groups (control and FMF) and M694V positivity-negativity in FMF group.

There was no statistically significant difference in the median E2, FSH, LH, PRL, and AMH levels (Figure 1) between the control and FMF groups (*P* > 0.05). Similarly, there was no statistically significant difference in either median right or median left ovarian volumes as well as either median right or median left ovarian follicle levels between the control and FMF groups (*P* > 0.05) (Table 2).

No statistically significant correlation was determined between age, gravidity, parity, number of abortions and children alive, BMI levels, number of siblings, age at FMF onset, age at FMF diagnosis, disease duration, and AMH levels in the FMF group (*P* > 0.05) (Table 3).

Distribution of mutation frequencies in decreasing order in the FMF group was M694V in 9 (27.2%) patients, E148Q in 7 (21.2%) patients, M680I in 7 (21.2%) patients, V726A in 4 (12.1%) patients, M694 M in 3 (9.3%) patients, P369S in 2 (6.06%) patients, and R761H in 1 (3.03%) patient.

In the FMF group, the median AMH level was statistically significantly lower in patients with M69V mutation than in those without M69V mutation (*P* = 0.018) (Table 4) (Figure 1). There was no statistically significant difference in median AMH levels between patients with and without E148Q mutation in the FMF group (*P* = 0.920) (Table 4).

There was no statistically significant difference in median AMH levels between patients with and without M680I mutation within FMF group (*P* = 0.856) (Table 4). No statistically significant difference was observed in the median AMH levels between patients with at least one mutation and no mutation in the FMF group (*P* = 0.868) (Table 4).

The mean white blood cell count (WBC) in FMF group was 6765.7 ± 2124.94 cells per microliter; MPV (mean platelet volume) was 9.6 ± 1.05 fL; mean ESR, CRP, fibrinogen, and vitamin D levels were 15.7 (11) mm/h; 9.5 (2) mg/dL; 294 ± 66.49 mg/dL; 19.4 ± 17.03 ng/dL, respectively. The mean disease severity score was 4.9 ± 2.07, and mean monthly attack frequency was 2.5 ± 1.96.

No statistically significant correlation was detected between AMH levels and WBC (*r* = −0.239, *P* = 0.167); MPV (*r* = 0.012, *P* = 0.945); ESR (*r* = −0.276, *P* = 0.108); CRP (*r* = −0.116, *P* = 0.506); fibrinogen (*r* = −0.239, *P* = 0.166); vitamin D (*r* = −0.169, *P* = 0.333); monthly attack frequency (*r* = −0.140, *P* = 0.422); and disease severity score (*r* = −0.123, *P* = 0.481).

## 4. Discussion

It is reported that FMF is most commonly encountered in North African and Iraqi Jews and Armenian, Turkish, and Arabic populations [1, 2]. In previous studies, its prevalence was determined between 1/150 and 1/10.000 [2]. Although FMF is encountered so frequently in some populations, its diagnosis may be delayed up to 7 to 8 years [2]. FMF attacks start spontaneously and last for at least 12 hours, and many of the symptoms disappear by themselves. In 90% of cases, the disease starts before 20 years of age, and it has been reported that the disease started before 10 years of age in 60% of cases [2]. Although the female/male ratio is reported to be similar, it has been shown in Turkey that FMF is observed a little bit more frequently in males (1.2/1) [9]. Many factors initiating and/or triggering the attack have been reported in patients with FMF [10]. It has been suggested that attacks can be set off by some causal agents, for example, emotional stress, disruption of treatment compliance, and the menstruation in females [10].

The Tel Hashomer criteria are commonly used in the diagnosis of FMF [8, 11]. There are also criteria, which were developed by Livneh et al. using Sheba Medical Center data and had a specificity of up to 99% [8]. However, the Tel Hashomer criteria are frequently used in practice. The most important point in FMF is that genotype does not reflect in the phenotype at the same rate in all patients [2, 12]. In other words, clinical signs may be different among individuals who have the same mutation, and more than 10% of patients may be mutation negative (not detected). It has been reported that disease may surface due to epigenetic mechanisms in these people [2].

In one study, it was reported that infertility rate was 1/3 among female FMF patients of reproductive age, who did not receive treatment [13]. It was proposed that the ovulatory dysfunction was due to inappropriate treatment [13]. In another study, it was indicated that ovulatory insufficiency was related to colchicine [14]. Again, it was reported that amyloidosis might be responsible for male and female infertility [15]. In a study in the literature, evaluating ovarian reserves in female FMF patients, inhibin, FSH, LH, E2, number of antral follicles, and ovarian volumes were determined on the 2nd–4th days of the menstrual cycles in both FMF patients and healthy women [16]. It was determined that ovarian reserves were decreased in FMF patients when compared with healthy individuals; however its mechanism could not be clearly defined [16]. History of drug use, disease severity, and MEFV gene mutation properties were not also considered in FMF patients in that study.

There are a few studies in the literature about the role of AMH levels in evaluating ovarian reserves in rheumatic diseases. Mont'Alverne et al. evaluated AMH levels and ovarian reserves in 10 patients with Behçet's disease and in 22 healthy controls [17]. In that study, AMH levels were markedly lower in patients with Behçet's disease (BD), whereas FSH levels were higher [17]. AMH levels were found to be markedly lower in patients with active disease, but the result was not statistically significant [17]. As a result, the study indicated that ovarian reserves were lower in the BD group when compared with healthy individuals [17]. However, limitations of the study were reported including a fewer number of patients and the cross-sectional design [17].

In our study on FMF patients, the number of patients was adequate when compared with the previous studies in the literature. However, our study was also designed as a cross-sectional study. There was amyloidosis in one of our patients, and the AMH level of that patient was lower (4.88 ng/mL). In this present study, no clear correlation was shown between the monthly attack number, disease severity score, and AMH levels (*r* = −0.140, *P* = 0.422 and *r* = −0.123, *P* = 0.481, resp.). Family history of dialysis was positive in three patients, and majority of the cohort was taking medication regularly (colchicine). In the literature, AMH levels were evaluated in female patients with Takayasu arteritis (TA), and it was shown that ovarian reserve decreased in one patient with TA [18]. Bleil et al. suggested that racial and ethnic differences may affect ovarian reserves [19]. This issue can be important and a confounding factor for our research cohort as well.

## 5. Conclusion

To the best of our knowledge this study is the first study assessing the ovarian reserves of FMF patients using AMH in addition to the hormonal parameters, ovarian volume, and antral follicle count (AFC) and determining its relation with disease severity and MEFV mutations. Additionally, we realized in the present study that there was no difference in ovarian reserves depending on AMH levels, AFC, and ovarian volumes between patients with FMF and healthy females. We believe that this finding might be due to our patient group characteristics. In our study, we determined that the AMH levels were lower in M694V mutation positive patients when compared with the M694V negative FMF patients. This can be due to the more severe inflammation and disease course that is expected in M694V positive patients. So in larger study groups including more severe FMF patients different results can be obtained. Limitations of our study may be indicated as inability to include patients in the attack and attack-free periods and with amyloidosis in the study and cross-sectional design of the study; and studies with long-term follow-ups might have been performed. If studies on larger FMF cohort with higher disease severity, resistant to treatment and with amyloidosis are performed, then different results may be obtained between AMH levels and ovarian reserves. Therefore, multicentered prospective studies are required to show ovarian reserves in an autoinflammatory genetic disease such as FMF and whether colchicine has favorable or unfavorable effects on this issue. The other side of the iceberg is not clear yet.

## Figures and Tables

**Table 1:** Demographic and clinical characteristics of the groups.

Variables	Control	FMF	*P* value
Age (yrs)	31.9 ± 6.1	31.7 ± 6.6	0.940^†^
Gravidity (*n*)	3 (0–7)	2 (0–6)	0.181^‡^
Parity (*n*)	1 (0–4)	1 (0–5)	0.116^‡^
Abortions (*n*)	0 (0–3)	0 (0–5)	0.751^‡^
Alive (*n*)	1 (0–4)	1 (0–5)	0.096^‡^
BMI (kg/m^2^)	24.7 ± 4.5	23.5 ± 4.6	0.080^¶^
Number of siblings (*n*)	3 (1–8)	4 (1–9)	0.115^‡^
Age at FMF onset (yrs)	—	18.5 ± 11.0	—
Age at FMF diagnosis (yrs)	—	24.5 ± 10.0	—
Disease duration (yrs)	—	6.5 (1–27)	—

^†^Student's *t*-test, ^‡^Pearson's chi-square test, and ^¶^Mann Whitney *U* test. BMI: body mass index; FMF: familial Mediterranean fever.

**Table 2:** Laboratory results, AMH levels, ovarian volumes, and antral follicle numbers of the groups.

Variables	Control	FMF	*P* value^†^
E2 (pg/mL)	39 (10–130)	52 (25–123)	0.136
FSH (IU/L)	4.8 ± 1.84	5.1 ± 1.95	0.561
LH (IU/L)	4.6 ± 2.23	5.4 ± 2.3	0.113
PRL (ng/mL)	9.8 (4.1–27.6)	11.2 (2.8–31.1)	0.711
AMH (ng/mL)	7.1 (4.6–11.0)	6.9 (4.9–10.8)	0.290
Right ovarian volume (cm^3^)	8.1 (4.3–29.7)	7.9 (3.0–28.7)	0.189
Left ovarian volume (cm^3^)	8.1 (4.8–23.5)	7.8 (2.3–11.3)	0.176
Right antral follicle number count (*n*)	9.1 (7.0–14.0)	8.8 (4.8–13.0)	0.276
Left antral follicle number count (*n*)	9.2 (8.0–15.0)	8.7 (3.9–10.0)	0.256

^†^Mann Whitney *U* test. FMF: familial Mediterranean fever; AMH: anti-Müllerian hormone.

**Table 3:** Correlation coefficients and their significance levels between AMH levels and demographic and clinical characteristics in the FMF group.

Variables	Correlation coefficient (*r*)	*P* value^†^
Age	−0.052	0.766
Gravidity	0.035	0.841
Parity	0.023	0.897
Abortus	0.059	0.736
Alive	−0.043	0.808
BMI	0.150	0.388
Number of siblings	0.077	0.661
Age at FMF onset	0.028	0.873
Age at FMF diagnosis	0.171	0.326
Disease duration	−0.200	0.248

^†^Spearman's rank correlation test.

**Table 4:** AMH levels according to mutations in the FMF group.

Mutation	*n*	AMH	*P* value^†^
M69V			**0.018**
Mutation negative	24	7.1 (4.9–10.8)	
Mutation positive	9	5.9 (5.4–7.1)	

E148Q			0.920
Mutation negative	26	6.9 (5.1–10.1)	
Mutation positive	7	6.7 (4.9–10.8)	

M680I			0.856
Mutation negative	26	6.8 (4.8–10.8)	
Mutation positive	7	7.0 (5.5–8.9)	

General			0.868
Mutation negative	9	6.9 (5.8–8.8)	
Mutation positive	24	6.8 (4.9–10.8)	

^†^Mann Whitney *U* test. AMH: anti-Müllerian hormone.
